# High Expression of COA6 Is Related to Unfavorable Prognosis and Enhanced Oxidative Phosphorylation in Lung Adenocarcinoma

**DOI:** 10.3390/ijms24065705

**Published:** 2023-03-16

**Authors:** Ming Zhang, Xiaohua Liao, Guanxu Ji, Xianming Fan, Qiang Wu

**Affiliations:** 1 Faculty of Chinese Medicine, Macau University of Science and Technology, Macao 999078, China; 2 State Key Laboratory of Quality Research in Chinese Medicine, Macao 999078, China; 3 Department of Respiratory and Critical Care Medicine, Affiliated Hospital of Southwest Medical University, Luzhou 646000, China

**Keywords:** cytochrome C oxidase assembly factor 6 (COA6), lung adenocarcinoma, prognosis, mitochondrion, oxidative phosphorylation

## Abstract

Mitochondrial metabolism plays an important role in the occurrence and development of cancers. Cytochrome C oxidase assembly factor six (COA6) is essential in mitochondrial metabolism. However, the role of COA6 in lung adenocarcinoma (LUAD) remains unknown. Here we report that the expression of *COA6* mRNA and protein were upregulated in LUAD tissues compared with lung normal tissues. We found that COA6 had high sensitivity and specificity to distinguish LUAD tissues from normal lung tissues shown by a receiver operating characteristic (ROC) curve. In addition, our univariate and multivariate Cox regression analysis indicated that COA6 was an independent unfavorable prognostic factor for LUAD patients. Furthermore, our survival analysis and nomogram showed that a high expression of *COA6* mRNA was related to the short overall survival (OS) of LUAD patients. Notably, our weighted correlation network analysis (WGCNA) and functional enrichment analysis revealed that COA6 may participate in the development of LUAD by affecting mitochondrial oxidative phosphorylation (OXPHOS). Importantly, we demonstrated that depletion of COA6 could decrease the mitochondrial membrane potential (MMP), nicotinamide adenine dinucleotide (NAD) + hydrogen (H) (NADH), and adenosine triphosphate (ATP) production in LUAD cells (A549 and H1975), hence inhibiting the proliferation of these cells in vitro. Together, our study strongly suggests that COA6 is significantly associated with the prognosis and OXPHOS in LUAD. Hence, COA6 is highly likely a novel prognostic biomarker and therapeutic target of LUAD.

## 1. Introduction

Lung cancer is a common malignant tumor worldwide. In 2020, more than two million new lung cancer cases occurred globally and the number of deaths was the highest among all cancers, up from 1.8 million cases [1]. Nonsmall cell lung cancer (NSCLC) accounts for about 85% of all lung cancers [2] and lung adenocarcinoma (LUAD) is the most common histological subtype of NSCLC [3]. Current therapies for LUAD include surgery, chemotherapy, radiotherapy, molecular targeted therapy, and immunotherapy [4]. LUAD patients usually lack symptoms in the early stage and many patients are in the middle and late stages when their symptoms appear. Hence, early diagnosis can significantly reduce the mortality of LUAD patients [5]. Although some progress has been made in the treatment of advanced patients, drug resistance often occurs, leading to treatment failure. In China, the 5-year survival rate of patients with lung cancer is less than 30% [6]. Therefore, identifying new biomarkers and therapeutic targets is important for the diagnosis and therapy of LUAD.

Mitochondrial metabolism is important for the occurrence and development of lung cancer. By using radiotracers and positron emission tomography to measure the mitochondrial membrane potential (MMP) in a mouse model of lung cancer, Momcilovic M et al. discovered that LUAD tissues possessed high MMP [7]. Analysis with data from The Cancer Genome Atlas (TCGA) indicated that the content of mitochondrial DNA in lung cancer tissues is higher than that in adjacent normal tissues [8]. Interestingly, metformin could reduce the production of cellular ATP by inhibiting mitochondrial respiratory chain complex Ⅰ [9]. In addition, in phase II clinical trial, metformin combined with an epidermal growth factor receptor-tyrosine kinase inhibitor significantly improved progression-free survival and overall survival (OS) of patients with advanced LUAD [10].

The cytochrome C oxidase assembly factor six (COA6) protein is a 14 kDa protein with a conservative motif CX9CXnCX10C. COA6 is mainly located in the inner mitochondrial membrane space. Importantly, COA6 is involved in the formation of cytochrome C oxidase (COX) [11]. Mutation or lack of COA6 can cause COX deficiency, adenosine triphosphate (ATP) synthesis disorder, and fatal neonatal cardiomyopathy [12]. These suggest that COA6 plays an important role in mitochondrial metabolism, yet the role of COA6 in LUAD remains unclear.

In this research, we found that COA6 was overexpressed in LUAD tissues. We then investigated the impact of COA6 on the diagnosis and prognosis of LUAD patients. Furthermore, we discussed the underlying mechanism of COA6 in LUAD. Finally, we verified the effect of COA6 on mitochondrial function and the proliferation of lung cancer cells in vitro.

## 2. Results

### 2.1. The Expression of COA6 Was Upregulated in LUAD

**Figure 1:**
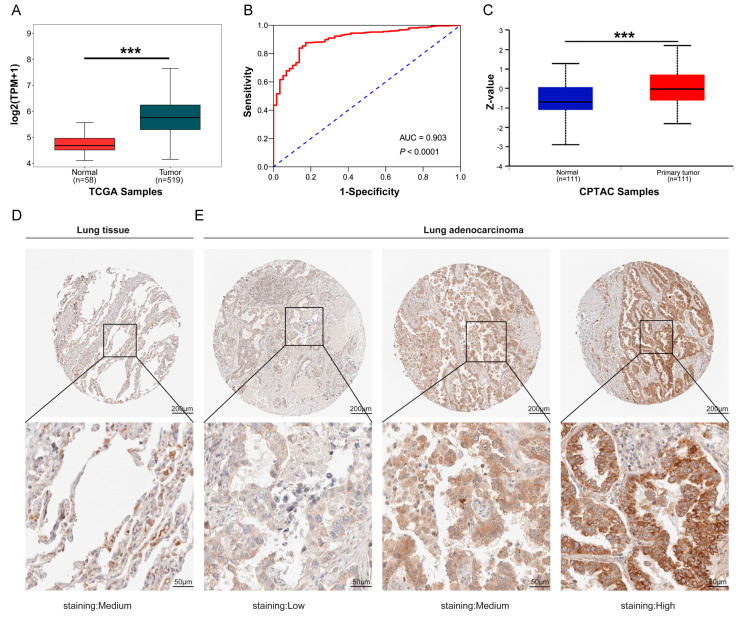
Expression of COA6 in LUAD and lung normal tissues. (**A**) *COA6* mRNA expression in LUAD shown in TCGA; (**B**) ROC curve showing the diagnostic value of COA6 in LUAD shown in TCGA; (**C**) COA6 protein expression of LUAD in the University of Alabama at Birmingham cancer data analysis Portal (UALCAN); (**D**,**E**) Immunohistochemical staining of COA6 in normal lung tissues and LUAD tissues in HPA. COA6 antibody was labeled with DAB (3,3′-diaminobenzidine), the brown color indicates the expression of COA6 protein, magnification: 50× (upper panel), 200× (lower panel). *t*-test of independent samples was used in comparing the means of two groups, *** *p* < 0.001.

To probe whether COA6 is a potential marker of LUAD, we first examined the COA6 expression in LUAD from multiple databases. We found that the level of *COA6* mRNA expression was higher in LUAD tissues than in normal lung tissues both in ONCOMINE and TCGA databases (*p* < 0.05) (Table 1 and Figure 1A). Our receiver operating characteristic (ROC) curve showed that COA6 had high sensitivity and specificity to distinguish LUAD and lung normal tissues in the TCGA database (area under the ROC curve (AUC) = 0.903, *p* < 0.0001) (Figure 1B). The level of COA6 protein expression was also higher in LUAD tissues than normal lung tissues in the Clinical Proteomic Tumor Analysis Consortium (CPTAC) database (*p* < 0.05) (Figure 1C). In the Human Protein Atlas (HPA), as LUAD tissues were from five patients, and lung normal tissues were from two patients, it is difficult to compare the expression of COA6 protein in LUAD tissue and normal lung tissue with statistical significance due to the small sample size. We also observed that COA6 was located in the cytoplasm and membrane. (Figure 1D,E and Table S1).

### 2.2. Associations of COA6 Expression Level with Clinical Parameters in LUAD

**Figure 2:**
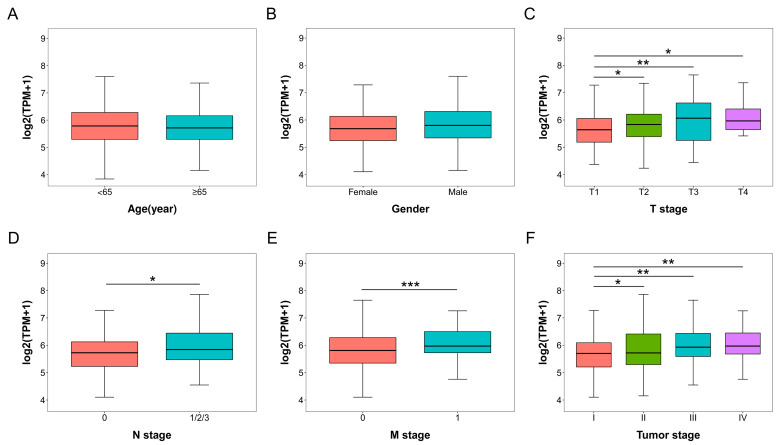
*COA6* mRNA expression in different clinical subgroups of LUAD patients in TCGA. (**A**) Age; (**B**) Gender; (**C**) T (the size of the tumor) stage; (**D**) N (lymph node metastasis) stage; (**E**) M (distant metastasis) stage; (**F**) Tumor (the size of the tumor, lymph node metastasis, and distant metastasis) stage. *t*−test of independent samples was used in comparing the means of two groups, * *p* < 0.05, ** *p* < 0.01, *** *p* < 0.001.

We then compared the *COA6* mRNA expression in different clinical subgroups of LUAD patients in TCGA (Table S2). In age and gender subgroups, there were no significant differences in *COA6* mRNA expression (Figure 2A,B). In the T (the size of the tumor) stages, the *COA6* mRNA expression was higher in T2, T3, and T4 than in the T1 groups (*p* < 0.05) (Figure 2C). In N (lymph node metastasis) stages, the *COA6* mRNA expression was higher in N1/2/3 than in N0 groups (*p* < 0.05) (Figure 2D). In M (distant metastasis) stages, the *COA6* mRNA expression was higher in the M1 than in the M0 groups (*p* < 0.001) (Figure 2E). In tumor (the size of the tumor, lymph node metastasis, and distant metastasis) stages, the *COA6* mRNA expression was higher in the II, III, and IV groups than the I group (*p* < 0.05) (Figure 2F). These data strongly suggest that the higher level of COA6 is related to the late stages of LUAD.

### 2.3. Prognostic Impact of COA6 Expression in LUAD

**Figure 3:**
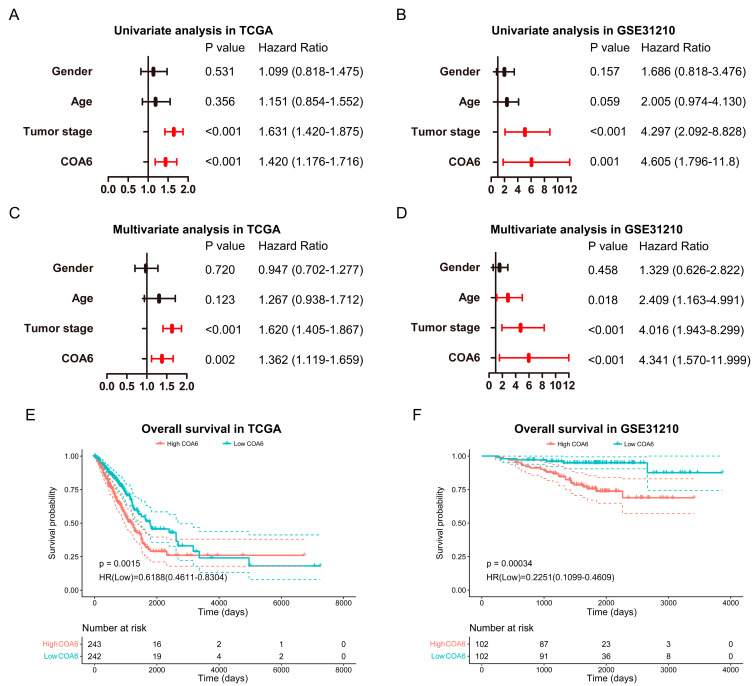
Impact of COA6 on the prognosis of LUAD patients. (**A**,**B**) Univariate Cox regression analysis in TCGA and GSE31210; (**C**,**D**) Multivariate Cox regression analysis in TCGA and GSE31210. (**E**,**F**) Kaplan–Meier survival curve for impacts of COA6 on overall survival of LUAD patients in TCGA and GSE31210.

**Figure 4:**
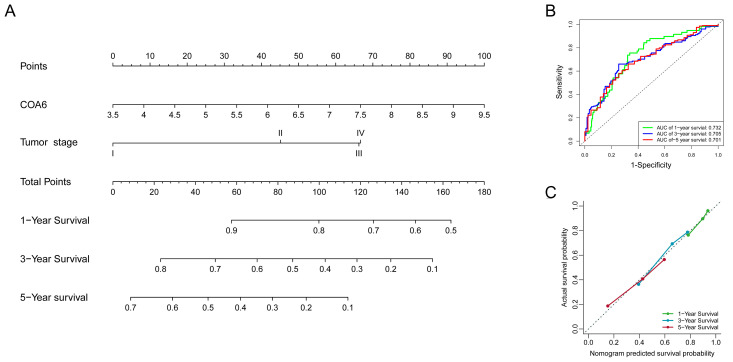
Prognostic model based on COA6 and tumor stage for LUAD patients in TCGA. (**A**) Nomogram based on COA6 and tumor stage. (**B**) ROC curve respecting specificity and sensitivity of nomogram for prognosis. (**C**) Calibration curve of the nomogram for overall survival at 1 year, 3 years, and 5 years.

To investigate whether COA6 expression can be a potential prognostic marker in LUAD, we performed bioinformatic analysis using datasets from TCGA (485 patients) and GSE31210 (204 patients) (Table 2). Our univariate and multiple Cox regression analysis of these two databases indicated that COA6 and tumor stage were independent unfavorable prognostic factors of LUAD patients (Figure 3A–D). Notably, our survival analysis showed that patients with a higher level of COA6 had a shorter OS (Figure 3E,F). In TCGA, we built a nomogram based on the two independent prognostic factors (COA6 and tumor stage) for OS of LUAD patients and found that the increase of COA6 is related to a reduction of the survival rate of patients (Figure 4A). Therefore, our ROC curve showed certain specificity and sensitivity of the nomogram for prognosis (AUC of 1, 2, 3-year survival: 0.732, 0.705, 0.701) (Figure 4B). Furthermore, our calibration curve showed a good prediction accuracy of the nomogram for one, three, and five-year survival (Figure 4C). The C-index of the nomogram based on COA6 and tumor stage was 0.684 (0.661–0.706), and the c-index of a nomogram based only on tumor stage was 0.664 (0.643–0.684). Taken together, we suggest that an increased COA6 level could be used to predict the prognosis of LUAD patients.

### 2.4. Difference of Biological Pathways between High and Low COA6 Groups in LUAD

**Figure 5:**
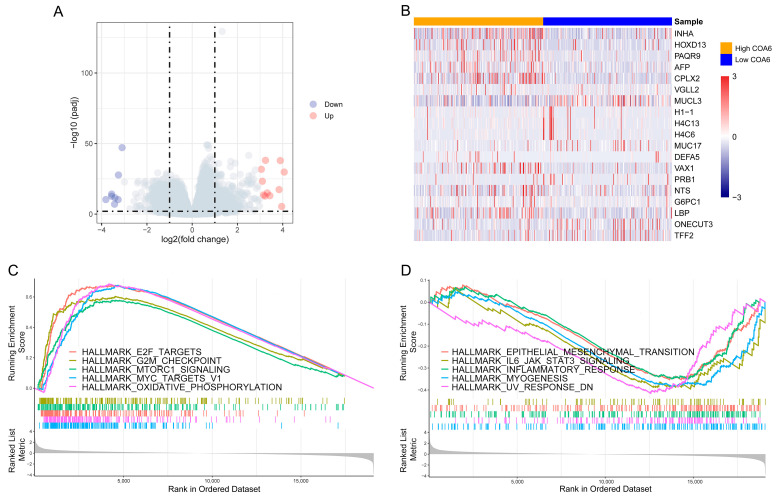
GSEA of DEGs between high and low COA6 groups. (**A**) Volcano map of DEGs; (**B**) Heatmap of DEGs with significant differences (*p*.adjust < 0.01, log2foldchange > 3). (**C**) The top 5 activated hallmark pathways in the high COA6 group; (**D**) The top 5 suppressed hallmark pathways in the high COA6 group.

Next, to identify the difference between biological pathways of high and low COA6 groups, we compared global gene expression differences between the two groups in TCGA. We found that there were a total of 19,101 genes with log2foldchange (Figure 5A,B and Table S3). We then performed a GSEA with the rank of log2foldchange. We discovered that there were 19 activated and 6 suppressed hallmark pathways in the high COA6 group if compared with the low COA6 group, (normalized enrichment score (NES)|>1, *p*.adjust < 0.05) (Figure 5C,D and Table S4). Notably, in the high COA6 group, the main activated pathways were OXPHOS, MYC targets, and mTORC1 signaling, whereas, the main suppressed pathways were UV RESPONSE DN, myogenesis, and IL6-JAK-STAT3 signaling.

### 2.5. Biological Pathways Correlated with COA6 in LUAD

**Figure 6:**
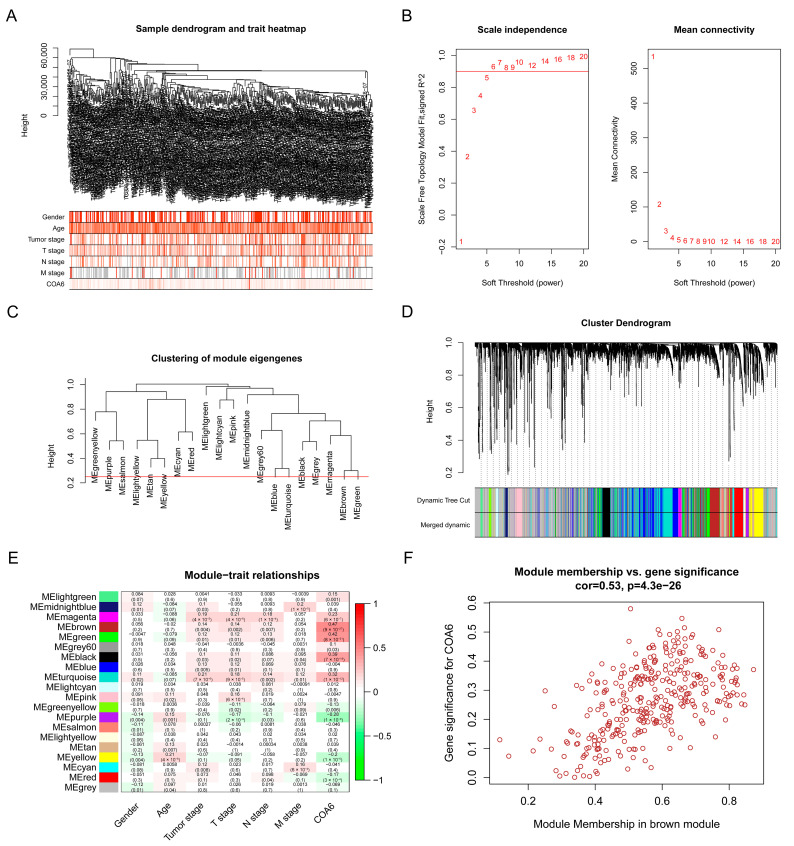
Screening for key modules related to COA6 in TCGA- LUAD through WGCNA. (**A**) Sample dendrogram and trait heatmap; (**B**) Calculation of the scale-free fit index of various soft-thresholding powers; (**C**) Clustering of module eigengenes. The red line indicates mergeCutHeight (0.25); (**D**) Clustering dendrograms; (**E**) Correlation heatmap between module eigengenes and clinical parameters and COA6; (**F**) Scatter plot of brown module eigengenes.

**Figure 7:**
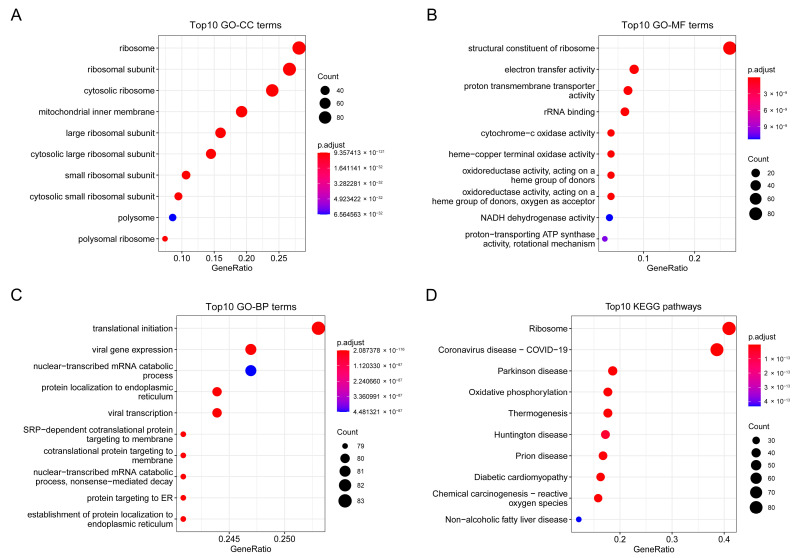
Function enrichment plots of genes in the brown module. (**A**) The top 10 enriched GO−CC terms; (**B**) The top 10 enriched GO−MF terms; (**C**) The top 10 enriched GO−BP terms; (**D**) The top 10 enriched KEGG pathways.

To find key module eigengenes related to COA6, we performed a WGCNA with the data of 485 LUAD patients from TCGA. We identified 4802 genes (top 25% of all genes according to variance) extracted by WGCNA (Table S5). We used clinical parameters and COA6 to construct a trait heatmap (Figure 6A). The soft threshold power was set as six (scale R^2 = 0.92) (Figure 6B) and mergeCutHeight was set as 0.25 (Figure 6C). We identified twenty modules (Figure 6D), in which the brown modules were most correlated with COA6 (Pearson coefficient = 0.47 and *p* = 9 × 10^−27^) (Figure 6E). We found 341 brown module eigengenes in a scatter plot (Figure 6F and Table S6). We also performed gene ontology (GO) and Kyoto Encyclopedia of Genes and Genomes (KEGG) pathway enrichment analysis with the brown module eigengenes. We found that these eigengenes were mainly correlated with the cellular component (CC) of the ribosome and mitochondrial inner membrane. Their molecular functions (MF) were focused on related electron transfer activity, cytochrome-c oxidase activity, and oxidoreductase activity. Moreover, the biological processes (BP) in which they participate included ribosome biogenesis and oxidative phosphorylation. Finally, the KEGG pathway closely associated with them contained ribosome, oxidative phosphorylation, chemical carcinogenesis-reactive oxygen species, and so on (Figure 7A–D and Tables S7 and S8). These data clearly demonstrate that COA6 plays essential roles in mitochondrial metabolism.

### 2.6. Down-Regulation of COA6 Inhibited the Proliferation of LUAD

**Figure 8:**
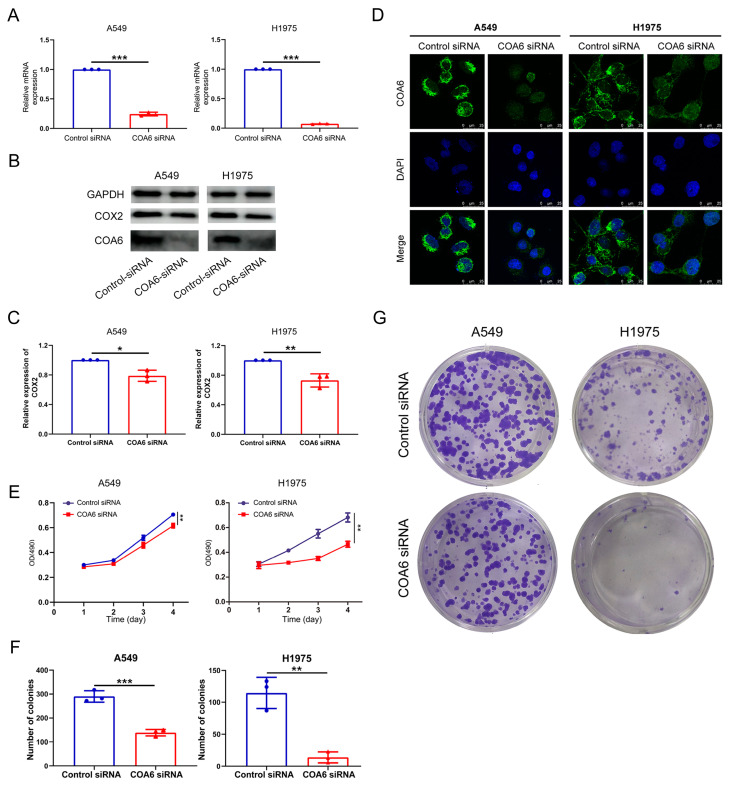
COA6 regulates the proliferation of LUAD cells (A549 and H1975). (**A**) Histogram showing the known down of *COA6* mRNA by siRNA (mean ± SD, n = 3); (**B**,**C**) Western blot showed that the decreased protein level of COA6 and COX2 by the knockdown of *COA6* (mean ± SD, n = 3); (**D**) Immunofluorescence staining showing the inhibition of the COA6 protein expression, Magnification: 400×; (**E**) MTT showing the inhibited proliferation of cells by the knockdown of COA6, the optical density (OD) values of two groups were compared at the 96th hour (mean ± SD, n = 3); (**F**,**G**) Colony formation assay showing the reduced number of colonies by the knockdown of COA6 (mean ± SD, n = 3). *t*−test of independent samples was used in comparing the means of two groups, * *p* < 0.05, ** *p* < 0.01, and *** *p* < 0.001.

To validate that COA6 is crucial in mitochondrial metabolism and LUAD, we used siRNA against *COA6* to knock down the expression of *COA6* in LUAD cells (A549 and H1975). Our qRT-PCR results showed that the COA6 mRNA level was significantly decreased by *COA6* siRNA (*p* < 0.001) (Figure 8A). Our Western blot further confirmed that the protein levels of COA6, together with COX2, were decreased by *COA6* siRNA in the LUAD cells (Figure 8B,C). In addition, our immunofluorescence affirmed that the expression of COA6 protein, which was mainly expressed in the cytoplasm in LUAD cells, was decreased upon *COA6* RNAi (Figure 8D). Importantly, our MTT analysis indicated that the knockdown of COA6 significantly reduced the proliferation rate of LUAD cells (Figure 8E). Our colony formation assays also showed that the knockdown of *COA6* could inhibit the formation of a colony of LUAD cells (Figure 8F,G). These results suggest that the knockdown of COA6 could decrease COX2 protein level and inhibit the proliferation of LUAD cells in vitro.

### 2.7. Down Regulation of COA6 Affected the MMP, Reactive Oxygen Species (ROS), and Nicotinamide Adenine Dinucleotide (NAD) + Hydrogen (H) (NADH) of LUAD

**Figure 9:**
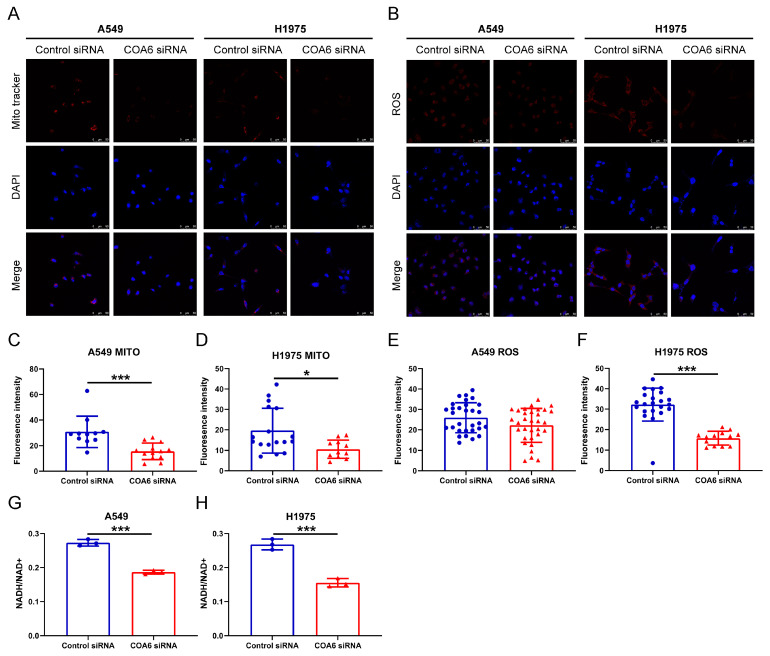
COA6 regulates the MMP, ROS, and NADH of LUAD cells (A549 and H1975). (**A**) Fluorescence staining showing the MMP affected by siRNA, Magnification: 200×; (**B**) Fluorescence staining showing the ROS affected by siRNA, Magnification: 200×; (**C**,**D**) The histograms showing that knockdown of *COA6* decreased MMP of LUAD cells (mean ± SD, C: n_control_ = 11, n_coa6_ = 13, D: n_control_ = 17, n_coa6_ = 11); (**E**,**F**) The histograms showing that knockdown of *COA6* did not decrease the ROS in A549 cells significantly, but decreased the ROS in H1975 cells significantly (mean ± SD, C: n_control_ = 30, n_coa6_ = 32, D: n_control_ = 22, n_coa6_ = 13); (**G**,**H**) The histograms showing that knockdown of *COA6* decreased NADH in LUAD cells (mean ± SD, n = 3). Independent samples *t*−test was used in comparing the means of two groups, * *p* < 0.05 and *** *p* < 0.001.

To further investigate the role of COA6 in mitochondrial metabolism, we measured mitochondrial membrane potential and intracellular ROS by the fluorescent probe. Our results indicated that knockdown of *COA6* significantly decreased the MMP of LUAD cells (*p* < 0.05) (Figure 9A,C,D) and also inhibited the production of ROS of H1975 cells (*p* < 0.05) since the ROS level in A549 cells was lower in the *COA6* siRNA group than in the control siRNA group, although without significant difference between the two groups (Figure 9B,E,F). We also observed that the NADH/NAD+ ratio in the *COA6* siRNA group was lower than in the control siRNA group (*p* < 0.001) (Figure 9B,G,H). These suggest that the knockdown of *COA6* can significantly reduce NADH production in LUAD cells.

### 2.8. Down Regulation of COA6 Deceased the Production of ATP in LUAD

**Figure 10:**
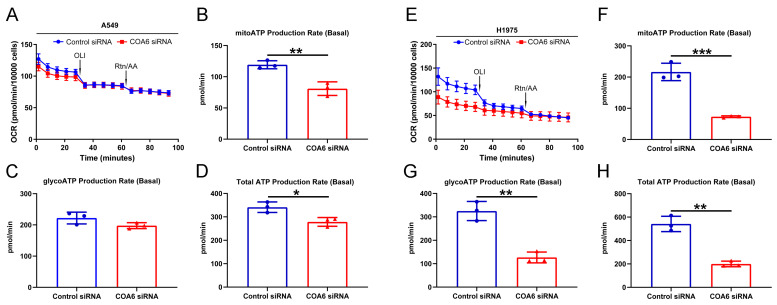
COA6 regulates the production of ATP in LUAD cells (A549 and H1975). (**A**,**E**) Line graphs showing the impact on OCR by OLI and Rtn/AA in different groups; (**B**,**F**) The histograms showing that knockdown of *COA6* decreased ATP produced by mitochondrial OXPHOS (mean ± SD, n = 3); (**C**,**G**) The histograms showing that knockdown of *COA6* did not decrease ATP produced by glycolysis significantly in A549 cells, but decreased ATP produced by glycolysis significantly in H1975 cells (mean ± SD, n = 3); (**D**,**H**) The histograms showing that knockdown of *COA6* decreased total ATP in LUAD cells (mean ± SD, n = 3). OCR, oxygen consumption rate; OLI, oligomycin; Rtn/AA, Rotenone/Antimycin A. Independent samples *t*-test was used in comparing the means of two groups, * *p* < 0.05, ** *p* < 0.01, and *** *p* < 0.001.

We performed a real-time ATP rate assay to uncover whether COA6 has a role in ATP production, We found that the total ATP and the ATP produced by mitochondrial OXPHOS were lower in the *COA6* siRNA group than in the control siRNA group (*p* < 0.05), whereas there was no significant difference of ATP produced by glycolysis between the two groups (Figure 10A–D). In contrast, in H1975 cells, the total ATP, ATP produced by mitochondrial OXPHOS, and ATP produced by glycolysis were all lower in the *COA6* siRNA group than in the control siRNA group (*p* < 0.05) (Figure 10E–H). These results suggest that the knockdown of *COA6* could decrease the production of ATP in LUAD cells in vitro.

## 3. Discussion

In this research, we first reported that both *COA6* mRNA and protein expression were overexpressed in LUAD and COA6 had high sensitivity and specificity to distinguish LUAD tissues and normal lung tissues. In addition, the expression of COA6 was correlated with the clinical parameters of LUAD patients. Our analysis indicated that COA6 is an independent risk factor for the prognosis of LUAD patients. Patients with a higher expression of COA6 had a shorter OS. These strongly suggest that COA6 is an important gene for the development of LUAD.

As a cytochrome C oxidase assembly factor, COA6 can interact with the copper-containing catalytic domain to maintain the stability of the newly synthesized COX subunit two (COX2) structure [13]. Loss of COA6 can lead to the rapid degradation of newly synthesized COX2. Subsequently, the level of COX decreases and the mitochondrial metabolism is impaired [14]. Since COX is essential for the process of OXPHOS as a terminal enzyme in the respiratory chain, a lack of COX usually leads to severe early-onset neuromuscular diseases and cardiomyopathy [15,16,17,18]. Current research on COA6 has mainly been concentrated on its role in mitochondrial metabolic diseases. Calvo SE et al. found that the mutation of COA6 was complicated by combined deficiency of mitochondrial complexes I and IV in the myocardial tissue of a patient with hypertrophic cardiomyopathy [19]. Subsequent studies confirmed that the deficiency of COA6 can disrupt COX assembly, reduce mitochondrial OXPHOS, and cause neonatal hypertrophic cardiomyopathy. Copper treatment can repair COX defects partially [12,20]. Recently, it has been proved that COA6 mainly acted as a thiol reductase in maintaining the structure’s stability of COX2, reducing the disulfide bond of the key cysteine residues in the copper metal chaperones (especially Sco1 and Sco2), and participating in the transport of copper [21,22]. Consistent with these findings, our results showed that the genes correlated with COA6 were mainly concentrated in the mitochondria, related to the activities of oxidoreductase, NADH dehydrogenase, and COX. In KEGG enrichment analysis, these genes were mainly related to oxidative phosphorylation. These strongly indicate that COA6 also has a crucial role in mitochondrial metabolism in LUAD.

Glycolysis was once considered to be the main metabolic pathway for the rapid proliferation of cancer cells, while mitochondrial metabolism was dispensable [23]. However, in recent years, research has proven that mitochondrial metabolism plays an important role in the occurrence and development of tumors. In mouse lung cancer driven by the *KRAS* gene, ROS, which is required for the growth of cancer, is mainly produced in mitochondrial metabolism and the deletion of mitochondrial transcription factor A could disrupt mitochondrial function and decrease tumor incidence [24]. In patients with lung cancer, tumor tissues had higher levels of glucose oxidation and tricarboxylic acid cycle than adjacent normal tissues [25]. In mouse lung cancer models, tumor tissues had a high MMP level, suggesting a high level of OXPHOS [7]. Decreased expression of heat shock protein family D member one could reduce mitochondrial OXPHOS, leading to inhibition of the growth of A549 cells [26]. Consistently, our research found that higher levels of *COA6* mRNA and protein in LUAD tissues and a large number of genes positively correlated with COA6 were enriched in the OXPHOS gene set, suggesting that COA6 is closely related to OXPHOS in LUAD. The OXPHOS pathway was significantly activated in the high COA6 group. These may explain why the expression of COA6 was associated with tumor stage and T stage in LUAD and patients with a higher expression of COA6 were of shorter OS. Our in vitro data also affirmed that the depletion of COA6 could decrease the mitochondrial membrane potential (MMP), NADH, and ATP production of LUAD cells, thus inhibiting the proliferation of these cells. Together, we propose that the uplifted COA6 level is closely related to the enhanced OXPHOS, which is beneficial to tumor growth and leads to the poor prognosis of LUAD patients.

Our gene-set enrichment analysis (GSEA) also showed COA6 is associated with MYC targets, ROS, mTORC1 signaling, glycolysis, E2F targets, DNA repair, and G2M checkpoints. As a transcription factor, MYC can enhance the growth and survival of lung cancer and other tumors [27,28,29]. Interestingly, MYC is important to activate mitochondrial biogenesis in cancer and could promote the growth of LUAD cells by regulating the structure and function of mitochondria [30,31]. Thus, we propose that COA6 is possibly involved in MYC-mediated tumor promotion. Intracellular ROS are mainly produced in mitochondrial metabolism and there are eight sites in the mitochondria that can produce superoxide [32,33]. ROS can activate the PI3K pathway by inhibiting PTEN (a PtdIns(3,4,5)P3 phosphatase) and increase the activity of AKT. Activation of the PI3K–AKT–mTOR pathway affects many cell biological processes, such as cell growth, proliferation, and differentiation [34,35]. For instance, activation of the mTOR pathway is related to the clinical outcome, invasiveness, and metastasis of LUAD [36]. Under hypoxia, ROS could activate hypoxia-inducible factors (HIFs) which can increase glycolysis and activate angiogenesis genes, and then promote the growth of the tumor [37,38]. In addition, E2F targets, DNA repair, and G2M checkpoints are important signal pathways in tumorigenesis and the development of LUAD [39,40,41]. Our result also showed that depletion of COA6 could decrease glycolysis-produced ATP and ROS in H1975 cells. Therefore, we suggest that COA6 may promote the proliferation of LUAD cells by multiple tumor promotion pathways including MYC, PI3K–AKT–mTOR, and ROS.

In conclusion, we demonstrated that COA6 is important in regulating OXPHOS. LUAD was significantly affected by COA6 and depletion of COA6 can inhibit OXPHOS and decrease ATP production, leading to inhibited proliferation of LUAD cells. Further clinical research can be carried out to affirm the functions of COA6 in mitochondrial metabolism in LUAD. Nonetheless, our COA6 study has provided novel insights into the roles of COA6 in cancer progression.

## 4. Materials and Methods

### 4.1. Bioinformatics Analysis

ONCOMINE [42] was used to compare the *COA6* mRNA expression of LUAD tissues and lung normal tissues. There were four datasets containing gene expression data of LUAD patients, respectively, from Stanford (Garber Lung [43]), Erasmus (Hou Lung [44]), Tokyo (Okayama Lung [45]), and Southern California (Selamat Lung [46]). UALCAN [47] was also used to compare their COA6 protein expression of them. The HPA [48] was used to obtain the immunohistochemical results of COA6 protein in LUAD and lung normal tissues.

To further analyze the relationship between COA6 and LUAD, we downloaded the RNA-seq and clinical data of LUAD patients from TCGA (https://portal.gdc.cancer.gov, accessed on 20 October 2021) and GSE31210 (http://www.prognoscan.org, accessed on 20 April 2022). RNA-seq data of 519 LUAD tissues and 58 adjacent normal tissues from TCGA were used to verify the results in ONCOMINE and plot the ROC curve. Patients’ inclusion criteria for clinically relevant analysis: the patients with RNA-seq data, complete clinical stages (T stage, N stage, M stage, and tumor stage), and survival data (OS and survival status). Then we compared the COA6 mRNA expression of different clinical subgroups in 485 LUAD patients from TCGA.

In TCGA and GSE31210, the impact of COA6 on patients’ OS was determined by univariate and multivariate Cox regression analysis. The patients were divided into a low COA6 group and a high COA6 group (the median of *COA6* mRNA expression was set as the group cutoff). Then, the OS of the two groups were compared by Kaplan–Meier analysis. A prognostic model was built using the independent prognostic factors of LUAD patients in TCGA through COX multivariate regression. The model was displayed using a nomogram. The specificity and sensitivity of the model were shown by the ROC curve. The accuracy of the model was evaluated by calibration curve and C-index.

Differentially expressed genes between the low COA6 and high COA6 groups from TCGA were used for GSEA, and hallmark gene sets (h.all.v7.4.symbols.gmt) were downloaded from the Molecular Signatures Database [49]. To find the module eigengenes most correlated with COA6, the data of 485 LUAD patients from TCGA were used for WGCNA. Then, functional enrichment analysis of these module eigengenes was performed.

### 4.2. Cell Culture and Transfection

LUAD cells (A549, H1975) were obtained from the cell bank of the Chinese Academy of Sciences. The cells were cultured in 1640 medium (GIBCO, Waltham, MA, USA), added with 10% fetal bovine serum (GIBCO, Waltham, MA, USA), and cultured in a humidified 5% CO_2_ incubator at 37 °C. lipornai™ transfection reagent (Beyotime, Shanghai, China) was used to transfect the control and *COA6* siRNA (Santa Cruz, Dallas, TX, USA) into cells. After 48 h from transfection, the effect of transfection was determined by qRT-PCR, western blot analysis, and immunofluorescence analysis.

### 4.3. RNA Isolation and qRT-PCR

Total RNA isolation, reverse transcription, and qRT-PCR were performed according to the product manual. The main kits included TransZol Up Plus RNA Kit (Transgen Biotech, Beijing, China), All-in-one First-Strand cDNA Synthesis SuperMix for qPCR (Transgen Biotech, Beijing, China), and Green qPCR SuperMix (Transgen Biotech, Beijing, China). The primer sequences for qRT-PCR were as follows: COA6: forward (5′-CAGTAGGAATGGCAGCCCCATCT-3′) and reverse (5′-CCCCCAGCAGACCTGTCTTTCCTT-3′); β-actin: forward (5′-CAGGGCGTGATGGTGGGCAT-3′) and reverse (5′-GATGCCGTGCTCGATGGGGT-3′).

### 4.4. Protein Extraction and Western Blot Analysis

Total protein was extracted using radioimmunoassay lysis buffer (RIPA, Thermo Fisher Scientific, Waltham, MA, USA). Protein assay dye reagent concentration (Bio Rad, Hercules, CA, USA) was used to measure the protein concentration. Western blot analysis was performed previously as described [50]. The primary antibodies were as follows: anti-COA6 (24209-1-AP, Proteintech, Wuhan, China), anti-COX2 (55070-1-AP, Proteintech, Wuhan, China), and anti-GAPDH (60004-1-Ig, Proteintech, Wuhan, China). Ultrasignal hypersensitive ECL chemiluminescence substrate (4A biotech, Suzhou, China) was used for chemiluminescence.

### 4.5. Cellular Immunofluorescence Assay

Cellular immunofluorescence assay was performed previously as described [51]. The cells were fixed with 4% formaldehyde and then infiltrated with 0.1% Triton X-100. Immunofluorescence stains of cells were performed with anti-COA6 antibody (24209-1-ap, Proteintech) and Alexa fluor 594 Goat antirabbit IgG (Thermo Fisher Scientific, Waltham, MA, USA). The nuclei were stained with 4′,6-diamidino-2-phenylindole.

### 4.6. MTT Assay and Colony Formation Experiment

Cells (1500 cells/well) were cultured in 96 well plates and transfected with siRNA. After 24, 48, 72, and 96 h, the cells were incubated with MTT solution for another 4 h. Cell viability was reflected by optical density (OD) at 490 nm. The transfected A549 and H1975 cells (1000/well) were seeded into 6-well plates. After 10 days, the cells were stained with crystal violet and the number of colonies was counted.

### 4.7. Detection of MMP, Intracellular ROS, and NADH/NAD+

Cells were cultured in 6 well plates and transfected with siRNA. After 72 h, MMP was labeled with Mito-Tracker Red CMXRos (Beyotime, Shanghai, China) and the intracellular ROS was labeled with DHE-ROS fluorescent probe (Bestbio, Shanghai, China). The cells were fixed with 4% formaldehyde and then infiltrated with 0.1% Triton X-100. The nuclei were stained with 4′,6-diamidino−2−phenylindole [52]. Cell number and fluorescence intensity were measured by Image-pro-plus. The intracellular NADH and NAD+ were detected with NAD+/NADH test kit (WST−8) (Beyotime, Shanghai, China). The concentration of NADH and NAD+ was reflected by optical density (OD) at 450 nm.

### 4.8. Real-Time ATP Rate Assay

Cells were cultured in 6 well plates and transfected with siRNA. After 48 h, plated A549 (12,000 cells/well) and H1975 (18,000 cells/well) in the Seahorse XFp Microplate, and 24 h later, Oligomycin and Rotenone/Antimycin A were used to affect the production of ATP. ATP rate was detected by Agilent Seahorse XFp according to the user manual. Cell number was estimated using Sulforhodamine B colorimetric assay [53].

### 4.9. Statistical Analysis

R (4.1.1) software and SPSS (25.0.0.0) software were used for statistical analyses, the main R packages used in the study were as follows: “ROCR”, “survival”, “survminer”, “DESeq2”, “clusterprofiler” and “WGCNA”. All in vitro experiments were repeated three times. A *t*-test and analysis of variance were used in comparing the means of two groups and multiple groups respectively, *p* < 0.05 means statistical significance.

## 5. Conclusions

In LUAD, COA6 is significantly correlated with the prognosis of patients and is important for mitochondrial metabolism. It is highly possible that COA6 is a novel prognostic biomarker and therapeutic target.

## Figures and Tables

**Table 1:** Significant difference between *COA6* mRNA expression in LUAD tissues and normal lung tissues (ONCOMINE).

Dataset	LUAD Cases	Normal Cases	Fold Change	*p*-Value	*t*-Test
Garber Lung	6	40	1.83	0.004	3.395
Hou Lung	65	45	1.698	5.33 × 10^−13^	8.652
Okayama Lung	20	226	1.673	3.08× 10^−12^	10.077
Selamat Lung	58	58	1.167	0.002	2.954

**Table 2:** Clinical data of the LUAD patients in TCGA and test GSE31210.

Clinical Factor	TCGA (n = 485)	GSE31210 (n = 204)
Survival time (days)	868.99 ± 872.11	1764.70 ± 683.65
Status		
Live	307	172
Dead	178	32
Age		
<65	216	145
≥65	269	59
Gender		
Female	262	109
Male	223	95
Tumor stage		
I	263	162
II	116	42
III	80	0
IV	26	0

## Data Availability

Not applicable.

## Supplementary Materials

The supporting information can be downloaded at: https://www.mdpi.com/article/10.3390/ijms24065705/s1.
