# Supplementary material for: High Expression of COA6 Is Related to Unfavorable Prognosis and Enhanced Oxidative Phosphorylation in Lung Adenocarcinoma
Source: Int J Mol Sci. 2023 Mar 16;24(6):5705. doi: 10.3390/ijms24065705 (PMC10056783; doi:10.3390/ijms24065705)
Supplement: Supplementary file 1 [file ijms-24-05705-s001.zip › Table S1.Immunohistochemical staining of COA6 protein in LUAD tissues and Lung normal tissues (HPA).pdf]

Table S1. Immunohistochemical staining of COA6 protein in LUAD tissues and Lung normal tissues (HPA)

Patient id	Gender	Age	Tissue type	Cell type	Staining	Intensity	Quantity	Location
1687	Male	65	Adenocarcinoma	Tumor cells	Medium	Moderate	>75%	Cytoplasmic/membranous
1847	Male	64	Adenocarcinoma	Tumor cells	Low	Moderate	<25%	Cytoplasmic/membranous
3003	Male	49	Adenocarcinoma	Tumor cells	Medium	Moderate	25%-75%	Cytoplasmic/membranous
3048	Female	67	Adenocarcinoma	Tumor cells	Medium	Moderate	>75%	Cytoplasmic/membranous
3052	Female	51	Adenocarcinoma	Tumor cells	High	Strong	>75%	Cytoplasmic/membranous
2101	Male	21	Normal tissue	Alveolar cells	Medium	Moderate	25%-75%	Cytoplasmic/membranous
				Macrophages	Medium	Moderate	>75%	Cytoplasmic/membranous
2268	Female	49	Normal tissue	Alveolar cells	Medium	Moderate	25%-75%	Cytoplasmic/membranous
				Macrophages	Medium	Moderate	>75%	Cytoplasmic/membranous
