# Supplementary material for: High Expression of COA6 Is Related to Unfavorable Prognosis and Enhanced Oxidative Phosphorylation in Lung Adenocarcinoma
Source: Int J Mol Sci. 2023 Mar 16;24(6):5705. doi: 10.3390/ijms24065705 (PMC10056783; doi:10.3390/ijms24065705)
Supplement: Supplementary file 1 [file ijms-24-05705-s001.zip › Table S2.COA6 mRNA expression in LUAD based on patientsí» clinical data.pdf]

Table S2. COA6 mRNA expression in LUAD based on patients' clinical data

Clinical parameters	Cases (n)	COA6 expression	P-value
Age (year)			
<65	216	5.823±0.771	0.487
≥65	269	5.777±0.696	
Gender			
Female	262	5.742±0.729	0.067
Male	223	5.863±0.728	
Tumor stage			
Ⅰ	263	5.687±0.681	0.001
Ⅱ	116	5.859±0.821	
Ⅲ	80	5.963±0.637	
Ⅳ	26	6.130±0.856	
Ⅰ vs Ⅱ			0.033
Ⅰ vs Ⅲ			0.003
Ⅰ vs Ⅳ			0.003
Ⅱ vs Ⅲ			0.318
Ⅱ vs Ⅳ			0.083
Ⅲ vs Ⅳ			0.305
T stage			
1	166	5.661±0.713	0.007
2	254	5.828±0.685	
3	44	6.014±0.973	
4	17	6.040±0.563	
1 vs 2			0.021
1 vs 3			0.004

1 vs 4			0.040
2 vs 3			0.115
2 vs 4			0.243
3 vs 4			0.901
N stage			
0	313	5.720±0.693	<0.001
1/2/3	161	5.971±0.771	
M stage			
0	319	5.843±0.735	0.034
1	25	6.172±0.846	
